# Detecting Deception in Movement: The Case of the Side-Step in Rugby

**DOI:** 10.1371/journal.pone.0037494

**Published:** 2012-06-11

**Authors:** Sébastien Brault, Benoit Bideau, Richard Kulpa, Cathy M. Craig

**Affiliations:** 1 M2S (Mouvement Sport Santé) Laboratory, University of Rennes 2 - Ecole Normale Supérieure de Cachan, Rennes, France; 2 School of Psychology, Queens University of Belfast, Belfast, United Kingdom; The University of Western Ontario, Canada

## Abstract

Although coordinated patterns of body movement can be used to communicate action intention, they can also be used to deceive. Often known as deceptive movements, these unpredictable patterns of body movement can give a competitive advantage to an attacker when trying to outwit a defender. In this particular study, we immersed novice and expert rugby players in an interactive virtual rugby environment to understand how the dynamics of deceptive body movement influence a defending player’s decisions about how and when to act. When asked to judge final running direction, expert players who were found to tune into prospective tau-based information specified in the dynamics of ‘honest’ movement signals (Centre of Mass), performed significantly better than novices who tuned into the dynamics of ‘deceptive’ movement signals (upper trunk yaw and out-foot placement) (p<.001). These findings were further corroborated in a second experiment where players were able to move as if to intercept or ‘tackle’ the virtual attacker. An analysis of action responses showed that experts waited significantly longer before initiating movement (p<.001). By waiting longer and picking up more information that would inform about future running direction these experts made significantly fewer errors (p<.05). In this paper we not only present a mathematical model that describes how deception in body-based movement is detected, but we also show how perceptual expertise is manifested in action expertise. We conclude that being able to tune into the ‘honest’ information specifying true running action intention gives a strong competitive advantage.

## Introduction

Perceiving biological motion is something we do quite naturally. Since the seminal work of Johansson [1] a number of studies have shown how, during an action, the relative movement of the points of light placed on strategic parts of the body can convey sufficient information to allow the perceiver to recognise the gender [2]–[6], the identity [7]–[10] but also the emotional state of an actor [11]–[12]. Furthermore, other aspects of non-verbal communication, such as action intention, can also be conveyed through the regularities of patterns of coordinated body movement and the relative movement of limbs [13]. Although the information embedded in the dynamic patterns of these unfolding actions can allow the perceiver to anticipate what the actor might do next, there are instances where the actor may want to disguise their true action intention [14]. This study will examine how deception is detected by expert and novice players in a rugby side-step and will show how the information embedded in the unfolding dynamics of the action influences expert and novice decisions about when and how to act.

In both natural and sporting duels, the movement of the body is used to deceive. Whether it is a cheetah chasing a gazelle in the Serengeti Park or a defender trying to catch an attacker on a rugby pitch, deceptive movement is used to gain a competitive advantage and beat an opponent. The side-step in rugby is an excellent example of how an attacker uses bodily movement to trick a defender into thinking they will run in one direction when they really intend to run in the opposite direction [15]–[16].

Jackson et al. [15] were the first to explore how expertise may affect ability to anticipate correctly the final running direction in a side-step in rugby. Using a temporal occlusion paradigm study they showed that expert players could accurately detect final running direction using significantly less information than novices [15]. Other studies have shown similar superior anticipatory skills related to expertise in basketball and handball [17], [18]. Although interesting to note these effects of expertise on perceptual performance, the studies to date fail to explain *what* information embedded in the unfolding pattern of body movement is being used to anticipate the resulting action intention.

**Figure 1:**
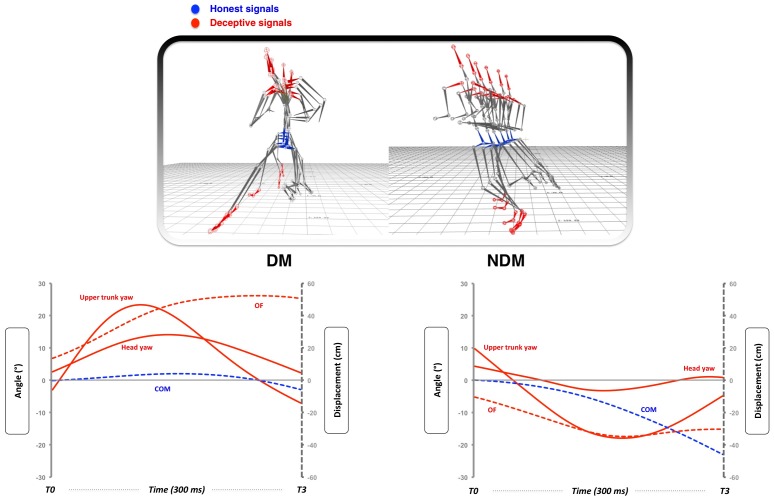
Dynamics of deception. The skeletal representations in the top two panels show how a deceptive (DM – left panel) and non-deceptive (NDM – right panel) movement unfold during the attacking player’s approach run. Each image represents a given moment in time during the unfolding movement and shows how the honest (blue) and deceptive (red) signals evolve during the movement. The graphs below show how during a deceptive movement the displacement of the honest signal (Centre of Mass (COM) displacement) is minimised whilst the displacement of the deceptive signals (i.e. Upper trunk yaw, Out Foot (OF) displacement and Head yaw) are all maximised. The non-deceptive movement (NDM) has a very different profile with all key body signals moving in a similar direction as the movement unfolds.

All purposive action, including deceptive movement, needs to be controlled ahead of time. Although much is known about how moving objects, governed by the laws of physics, are intercepted [19]–[20], little is known about how moving people or animals, intentionally controlled by independent nervous systems are caught. How, in these instances, can patterns of body movement prospectively inform a predator or defender about the future course of action of their target, and how does the unfolding action signal deception to the observer? In an attempt to understand how the unfolding action coveys deception, Brault and colleagues analysed the biomechanical differences between deceptive and non-deceptive movements [16]. They showed how deception is conveyed by exaggerating the movement of certain parts of the body (out-foot placement, head and upper trunk yaw) that are *not* mechanically related to the final running direction. Interestingly, they also showed that movements of parts of the body that *are* related to final running direction (i.e. Centre of Mass (global body) displacement and lower trunk yaw) need to be minimised to ensure the player can still change the angle of the run (see Figure 1). This difference between exaggerated and minimised body based movement essentially determines the success of a deceptive movement [16]. From an evolutionary biology perspective exaggerated body movements can be thought of as conveying deceptive signals while the minimised body movements can be thought of as conveying honest signals [21].

Although previous studies have detailed the biomechanics of deceptive movement or shown superior perceptual judgments [16], [22] in expert performance, they have tended to neglect the role that prospective, perceptual-based information, specified through the unfolding pattern of body movement, plays when making perceptual judgments. Furthermore they also fail to show how prospective information embedded in the unfolding action influences decisions about when and how to act. In other words, they do not show how perceptual information guides the temporal unfolding of an action. The solution presented in this study addresses these two issues. In two different experiments we will analyse deceptive movements in terms of the timing and control of the unfolding action using state of the art immersive, interactive virtual reality technology. In the first experiment we will attempt to identify what perceptual information is picked up and used by the perceiver to anticipate the attacking player’s action intentions. In a second experiment we will use these findings to make predictions about how players (novice and expert) should respond when faced with a virtual side-stepping attacker and test these predictions through an in-depth analysis of movement.

**Figure 2:**
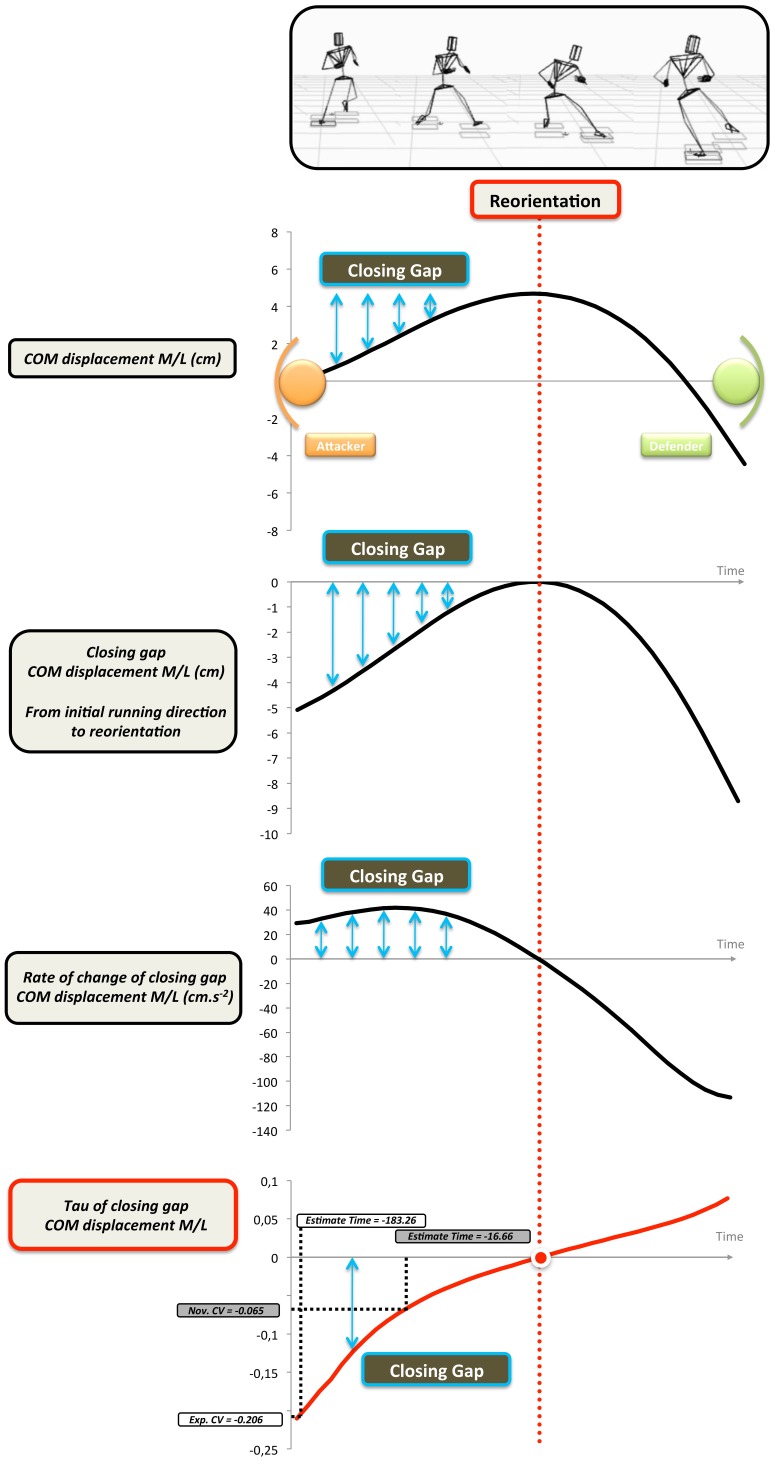
Example of the Tau COM displacement medio-lateral (M/L) for a DM to the right. This figure shows an example of how Tau of closure of the COM M/L displacement gap is calculated to detect the point of reorientation. In this example, the closing motion-gap (top panel) is defined as the difference between the initial running direction and the maximal medio-lateral displacement (which corresponds to the reorientation peak symbolized by the vertical red dotted line). For the other angle parameters, such as upper trunk yaw, the same procedure is used but the gap is closed from the initial orientation before the DM (straight run ∼0°) to the point of maximal orientation (i.e. the reorientation peak for this parameter). The middle panel shows the rate of change of the COM displacement towards the point of reorientation and the bottom panel shows the tau of the COM displacement. On the bottom graph the critical values (CVs) are presented for both experts (Exp. CV) and novices (Nov. CV). These values represent the time when the information becomes most important. Note how it is much sooner for the experts than the novices.

The model we present to capture the dynamics of the unfolding action is derived from tau theory [23]–[25]. Tau is a dynamic property of the environment actor system that encapsulates how a motion-gap, that can be a distance, angle or force, changes over time. This invariant property related to the dynamics of an event provides prospective information and is simply defined as the ratio between current motion-gap size, *x,* and its current rate of closure, 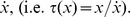 Although this invariant has been reliably shown to prospectively guide action when projectiles are intercepted [26] or struck [25], this study investigates how this temporal-based invariant guides action when the information involves observing biological movement. In this study, the gaps are defined as the difference between current positions or angles of body segments and the final end-points at body reorientation (see Figure 2).

Through our analysis we will see how the dynamics of the relative movement of different parts of the body with respect to the point of body reorientation (Experiment 1) can guide the defender’s future course of action (Experiment 2). Depending on whether the defender attends to the ‘honest’ or ‘deceptive’ signals conveyed through the movement of relative body parts, this model of deception should theoretically explain which action a defender will choose to make (e.g. move left or right to catch the player). By extending the model further we can show how the defender can use this perceptual information (tau) to temporally guide the closure of the gap between himself and the attacking player so he successfully intercepts the player (see Figure 2) in a similar way to catching an object in a goal zone [26].

In the first experiment, a Perception Only task, we look at how prospective (tau) information embedded in the unfolding patterns of honest and deceptive signals during a rugby side-step can influence a player’s judgment about final running direction. We also examine the role of expertise. In the second experiment, a Perception and Action task, we attempt to relate the findings from experiment 1 to make predictions about how an expert and novice player should act when confronted with a virtual side-stepping attacker. In other words, we explore the relationship between perceptual expertise and the dynamics of the ensuing action. By coupling perception to action we are able to not only show how perceptual information influences decisions about when and how to act but also show how the defender can use perceptual information picked up from the dynamics of the attacking player’s movements, to control his actions to intercept the player.

## Methods

### Experiment 1: Perception Only

#### Participants

Fourteen expert rugby players (M = 23.4 years; SD = 2.3 years) and 14 non-rugby players (M = 22.6 years; SD = 3.3 years) took part in the study. All expert players were professional rugby players competing regularly in top-level European competition. All had international experience (mean playing experience  = 13.3 years; SD = 5.6 years). All novices were students at the university and had no experience playing rugby. The study was approved by the local ethics committee and adhered to the standards laid down in the Declaration of Helsinki. All participants gave written informed consent before participating.

#### Immersive interactive virtual reality

This study used state of the art immersive, interactive virtual reality technology as a means of presenting perceptual information to the participants and measuring their responses. This novel technique has now been successfully used in several different sporting contexts including football and handball [27], [28]. The advantages of this technology over traditional methods such as videos or image stills are that i) the viewpoint of the unfolding action is player centred (as in a real-life setting), ii) there is complete control over the information presented to the player and iii) the actions recreated are full 3 dimensional representations of real captured movements. Furthermore the simultaneous recording of the action responses and the approaching avatar mean that direct links can be made between the perceptual information and the ensuing action.

#### Display and tracking

Participants viewed the virtual rugby stadium through two small screens inside a stereoscopic head mounted display (HMD) (Cybermind Visette 45™, resolution 1280*1024, diagonal field of view 45°). To give a feeling of 360-degree immersion an Intersense wireless (IS 900) head tracker was mounted on the front of the headset (InterSense Inc., Bedford, Massachusetts, USA) and was used to update in real time (120 Hz) the egocentric viewpoint (displacement and rotation) projected inside the headset. The volume within which the tracker could be tracked was 6 m wide by 8 m long by 3 m high. The control box for the HMD was housed in a wooden and aluminium case which was mounted on a backpack with adjustable straps. Two 8 m DVI cables connected the HMD control unit to the computer.

#### Creating virtual side-steps from real actions

Instead of using videos of stepping actions that do not involve real defenders [15], here we used real-life 3D motion capture recordings of a real attacker trying to step and beat a real defender. This rich source of data not only allows us to select effective deceptive movements [16] but it also allows for more realistic animations of the virtual attacker and deceptive movements in the immersive, interactive virtual rugby setting.

**Figure 3:**
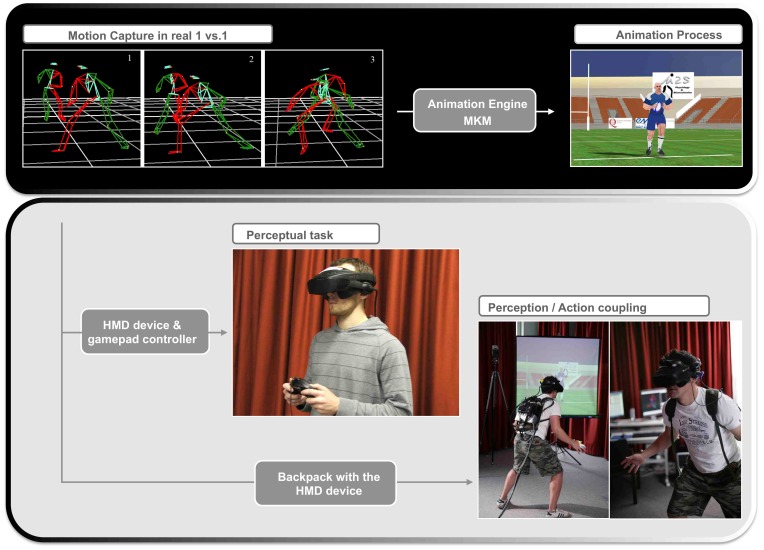
Top panel. Real movement data recorded from attacker versus defender duels are used to form the basis of the movement of the animated virtual attacker. Bottom panel: The two immersive tasks in a virtual rugby environment used a Head Mounted Display with a wireless motion tracker. This gave the participants a fully (360 degree visual field) immersive experience. In the Perception Only task, a gamepad was used to record the participant’s predictions about final running direction (by pressing left or right buttons) at the different occlusion times. In the Perception and Action experiment, participants wore a backpack containing the control unit for the HMD so that they not only had a fully immersive experience but that they were also free to move (up to 3 m to the left or right) to intercept the virtual attacker. Their movements were recorded using the Qualisys motion capture system.

Eight French national league rugby players (mean age 21.38 years; SD = 1.18 years) took part in these real attacker vs. defender duels. Following the recommendations of the International Society of Biomechanics (ISB) [29]–[31], both the attacker and defender wore 38 reflective markers at key anatomical landmarks on the body. Movement of both players was recorded using the optoelectronic motion capture Vicon MX system (Oxford Metrics, Oxford, UK) (Figure 3 and Video S1). The attacking player was asked to try and beat the defender, by performing either a side-step (deceptive movement (DM)) or simply running past the defender (non-deceptive (NDM) movement) [16].

The efficacy of the attacker’s deceptive movement was determined by analysing the defender’s response. Eight different deceptive movements (DMs) that caused the defender to move a minimum of 5 cm (Centre of Mass (COM) lateral displacement) in the opposite direction to the final running direction were selected [16]. Four involved the attacker faking a movement to the right before passing to the defender’s left and four involved faking a movement to the left before passing on the defender’s right. The other four attacking runs (NDMs) were made up of two simple directional changes: two to the left and two to the right of the defender. The attacking players’ movements recorded during these sessions formed the basis of the animation of the virtual rugby player. No social cues (e.g. facial expressions or eye movements) were recorded or used in the animation process.

Although the biological motion of the real rugby players formed the basis of the virtual rugby player, certain adaptations needed to be made to ensure the movements were credible. The animation engine MKM (Manageable Kinematic Motions) [32] (Figure 3 and Video S2), which provides a framework combining several adaptation modules to ensure the overall pattern of motion is not altered, was used to adapt the morphology of the real rugby players to that of the virtual rugby player. This meant that important events, such as foot contact with the ground during the stance phase, were simulated accurately to recreate realistic character movement. This software has already been used and validated in other movement simulations that involve other types of sporting duels [33]. The 3D development software Virtools 4.0 (Dassault Systemes, Paris, France) was then used to manage and integrate all the different developed components that make up the virtual rugby environment. This included the rendering of the 3D rugby pitch, the playing of the humanoid animation (via MKM), the management of the interface with the head tracker, reading in the data from the head tracker and using this to update, in real time, the egocentric viewpoint of the player in the virtual world.

#### Conditions

**Figure 4:**
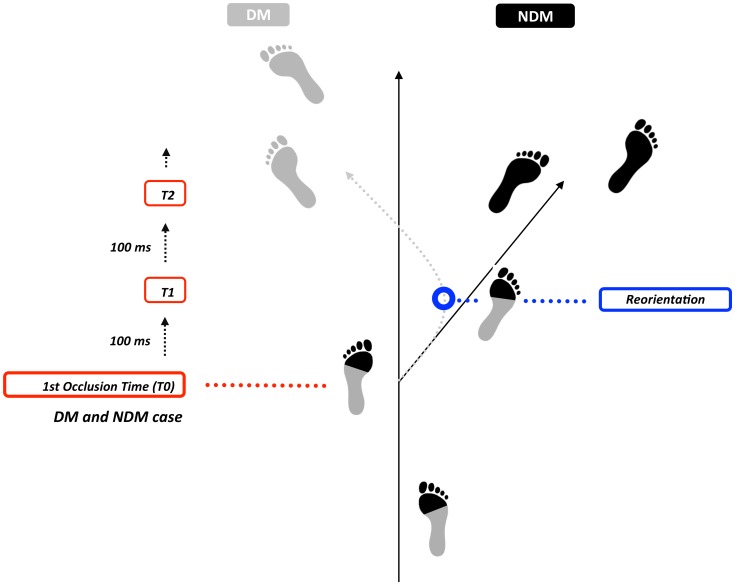
Protocol for Determining Occlusions. Footstep patterns for a Deceptive Movement (DM - grey - Movement towards the right, reorientation back towards the left) and a Non-Deceptive Movement (NDM - black - Movement towards the right with no reorientation). The first occlusion time (T0) is defined as the moment the attacker’s foot makes contact with the ground before the first directional change in the movement (towards the right in this instance).

**Figure 5:**
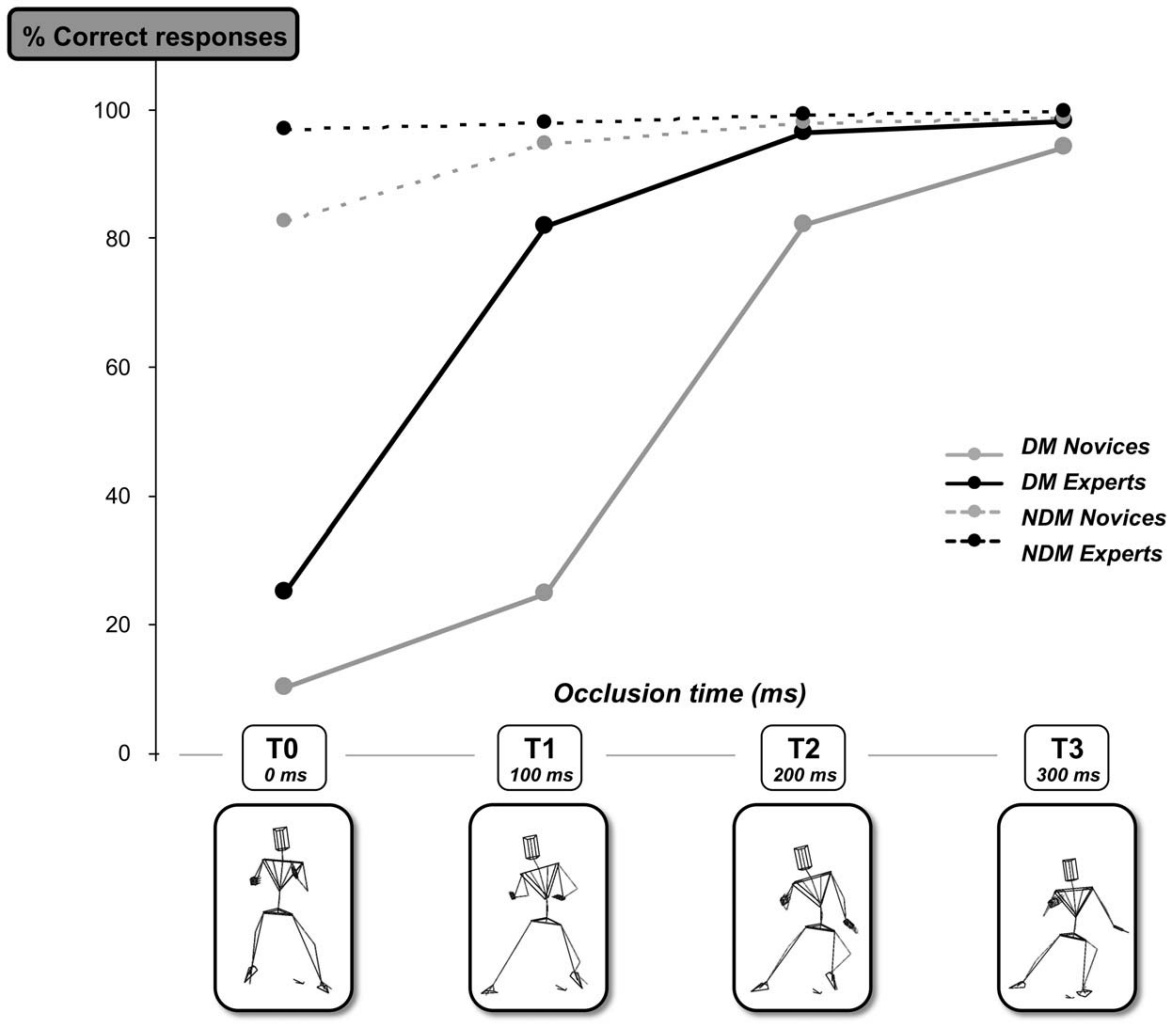
Overview of correct responses for both Novice and Expert participants. Mean percentage of correct responses for both novice (grey line) and expert (back line) groups when presented with deceptive (DM – solid line) and non-deceptive (NDM – dashed line) movements at the four different occlusion times (T0, T1, T2 and T3). The stick figures below represent the differences in static body configuration at each occlusion time.

Conditions were created based on the occlusion time paradigm. This well tested method allows us to understand how the quantity of visual information (in successive occlusions) influences decisions about the future course of action. The spatial reference point for the first occlusion (T0) was taken as the moment the attacker’s foot made contact with the ground during the footfall before reorientation (Figure 4). The other occlusion times were taken at 100 ms (T1), 200 ms (T2) and 300 ms (T3) later. As the unfolding pattern of movement of DMs and NDMs was similar at the beginning of the attacking run, it was predicted that players will more accurately judge the final running direction for DMs as more visual information becomes available (Figure 3 and Figure 5). As there is no body reorientation phase for NDMs, the predictions should stay the same throughout the movement (Figure 3 and Figure 5).

#### Task

Participants wore a stereoscopic Head Mounted Display (HMD) with attached head tracker (Video S2). To assess their ability to judge a virtual attacking player’s final running direction, the participants took on the role of defender. Twelve different attacking runs (8DM and 4NDM) (Video S1) were occluded at four different time points (the footfall before the first orientation initiated by the attacker and three successive 100 ms steps thereafter (Figure 4 and Methods)). Following a short familiarisation period, participants were asked to judge, after stimulus presentation, the final running direction of the attacker (left or right) by pressing the corresponding button on the gamepad (Figure 3). Two hundred and forty movements ((8 DM +4 NDM) * 4 occlusions * 5 repetitions) were pseudo-randomly presented. Pauses for rest were given after blocks of 60 trials.

#### Analysis methods – invariant information in deceptive movement

Although other studies have alluded to the importance of relative movement between limb segments [15], [34], none have examined *what* the invariant information is that we could use to make prospective judgements. Here we propose that one potential variable that could allow players to accurately detect a reorientation in body alignment in a deceptive movement (and as a result a change in running direction) could be ‘Tau’ [23] – a spatio-temporal variable that encapsulates not only the magnitude of a motion-gap (distance, angle or force) but its current rate of closure. As mentioned previously, the gaps are defined as the *difference* between current positions or angles of body segments and the final end-points at body reorientation. From Figures 1 and 2 it can be observed that both the chronology and magnitude of gap closure of different body parts used to signal ‘honest’ and ‘deceptive’ information are different, hence the taus, specifying the time to gap closure for each signal will also be different.

To examine this hypothesis we calculated the tau of both the deceptive (Out-Foot (OF) placement, head and upper trunk yaw) and honest signals (COM displacement). The tau of each variable was calculated as follows:
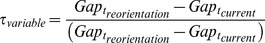
(2)where 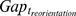
corresponds to the magnitude of the final motion-gap at the time when the reorientation point is reached (*t_reorientation_*) and 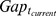
corresponds to the magnitude of the current motion-gap at a given moment in time for the different kinematic parameters (*t_current_*). The denominator is the 1^st^-order differential with respect to time of the motion-gap specified in the nominator. As the final motion-gap is taken as being the moment the gap is closed it is considered as being zero. The gap size, as specified above, will therefore continually decrease until it equals 0 (cm or °) at the point of reorientation. This formula is used for both deceptive (OF medio-lateral displacement and head and upper trunk yaw) and honest (COM medio-lateral displacement) signals and informs about the time remaining before the reorientation point of each parameter is reached.

Figure 2 shows an example of how the tau of the closure of the COM M/L displacement gap is calculated with respect to the point of reorientation. In this example, the closing motion-gap is defined as the difference between the initial running direction and the maximal medio-lateral displacement (which corresponds to the point of reorientation). For the other parameters, such as upper trunk yaw, that represent angular changes, the same procedure is applied. In this case the size of the gap is calculated as the difference between the initial angle before the point of reorientation (straight run ∼0°) and the maximum angular change at the point of reorientation.

We hypothesise that players not fooled by a deceptive movement, mostly experts, will tune in earlier to the honest signals (i.e. COM medio-lateral displacement) that specify true running direction explaining their superior performance in judging final running direction. As a comparison, we also considered other potential informational variables namely the magnitude of a gap (*x*), the rate of change of this gap 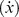 as well as the Tau of the gap (*τ_x_*) at the four different occlusion times. The yaw of the lower trunk, which represents pelvic movement, did not show any regular reorientation pattern [16], and was therefore not analysed. By regressing the percentages of correct responses for the two different groups of participants (expert and novice) onto each information variable and fitting a logistic (S-shaped) function to determine the goodness of fit (‘a’ and ‘b’ are constants, ‘u’ is the upper bound), the strength of the relationship for a particular information variable can be determined.
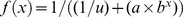
(1)


All variables and their corresponding R^2^ values are presented in Table 1.

**Table 1:** Differences in strategy and sensitivity to different information variables.

				R^2^	Estimate Time (ms)
Honest Signal	COM displacement M/L (cm)	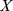	Exp.	0,01	
			Nov.	0,02	
		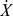	Exp.	0,00	
			Nov.	0,00	
		**Tau**	**Exp.**	**0,74**	**−183,26**
			**Nov.**	**0,51**	**−16,66**
Deceptive Signals	Head Yaw (°)	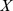	Exp.	0,00	
			Nov.	0,01	
		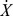	Exp.	0,03	
			Nov.	0,05	
		**Tau**	**Exp.**	**0,52**	**−158,26**
			**Nov.**	**0,60**	**−16,66**
	Upper Trunk Yaw (°)	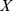	Exp.	0,08	
			Nov.	0,03	
		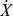	Exp.	0,01	
			Nov.	0,41	
		**Tau**	**Exp.**	**0,54**	**−141,66**
			**Nov.**	**0,67**	**66,67**
	OF displacement M/L (cm)	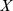	Exp.	0,00	
			Nov.	0,01	
		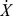	Exp.	0,00	
			Nov.	0,00	
		**Tau**	**Exp.**	**0,51**	**−283,33**
			**Nov.**	**0,44**	**−183,34**

The left hand side of the table shows how the information used differs between experts and novices as shown by different coefficients of determination (R^2^) for the honest (COM displacement M/L) and deceptive (Head Yaw, Upper Trunk yaw and OF displacement M/L) signals. The right hand side of the table highlights differences in the sensitivity to the different signals between groups as shown by the information pick up time estimates (ms) derived from the logistical regression critical values (CV) for all parameters. The last column on the right shows the differences in time (ms) between the two groups. Note how the experts are picking up information earlier than the novices.

#### Critical value & estimate times

Given that the pattern of body movement is different for deceptive and non-deceptive movements, we predict that the deceptive signals will be used more by the novices and the honest signals will be used more by the experts. The extent to which a signal is utilised is manifested by the strength of the coefficient of determination (R^2^), with higher values indicating a greater percentage of the variance in response accuracy being explained by that particular variable or signal. From the logistic equations used to calculate the coefficients of determination, we can also derive the critical values (CVs) or threshold points where the percentage of correct answers exceeds 50%. In order to estimate the time when the percentage of correct responses are greater than 50%, the CVs are repositioned on the mean curve for each parameter (Figure 2). As tau is a temporal variable, a critical value would provide an indication of the time when a player picked up the relevant information to correctly judge final running direction. These estimated values for the tau variable can therefore provide a means of discriminating between the time when information pertaining to a given signal is being picked up, highlighting a participant’s sensitivity to that variable. The results for these time estimates are presented for both honest and deceptive signals in Table 1.

### Experiment 2: Perception and Action

Although parallels in perceptual expertise and associated neural correlates have previously been shown when reading body kinematics [21], the similarities between perceptual expertise and the dynamics of expert action have not. Furthermore, decoupling perception and action has often been criticised as being too far removed from the real task, with some studies even suggesting the activation of different neural pathways [35]. In an attempt to address these issues, we extended the protocol presented in experiment 1 in a second experiment where perception and action were coupled. Instead of limiting the presentation of information by cutting off the displays and asking participants to judge what would happen next, we allowed participants to respond as they would in a natural setting, that is move as if to ‘intercept’ the virtual attacker. By allowing the participants to move in response to the information presented in the HMD, we can understand *how* perceptual information, in the unfolding event, informs decisions about *when* and *how* to act. We can also closely examine how the defender uses the temporal unfolding of the dynamics of the attacking player’s movements to guide their own actions.

In this experiment, we hypothesise that the perceptual information picked up by the defender during an attacking player’s approach run will influence their movement responses. We also predict that players showing perceptual expertise in reading invariant ‘honest’ body kinematic signals will also be experts when it comes to action choice and action control. In other words expert players who tune into the information specifying true running direction will perform better than novices in the following ways. Firstly, as a deceptive movement involves exaggerating certain body based movements to try and signal early a false running direction, we predict that the novice players, who are more susceptible to this deception, will initiate their movements much earlier than experts. Secondly, expert players who tune into the honest signals will be less fooled by deceptive movements and will therefore make fewer initial movements in the wrong direction (movement biases) when trying to ‘intercept’ the attacking player. And thirdly, as the task involves picking up perceptual information that will allow the defender to anticipate where the player will go, we predict that the final distance between the virtual attacker and the real defender will be less for the experts. In other words they will be more successful at anticipating the final running direction and stopping the virtual attacker.

**Figure 6:**
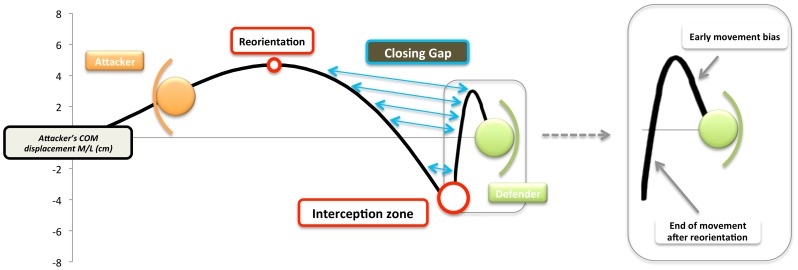
Relationship between the displacement of the attacker and a novice defender. This schematic diagram shows the reorientation point for the attacker and the distance gap that needs to be closed so that the defender can intercept the attacker (blue arrows). The interception zone shows where this took place. The panel on the right shows an early movement bias, that is a movement in the wrong direction caused by the deceptive movement of the attacker. The tau-coupling analysis looked at how the information embedded in the movement kinematics of the attacker from the point of reorientation to the interception zone (tau perception), influenced the way the defender moved to close the gap between them and the attacker (tau action).



In an attempt to see how much the perception of the dynamics of the movement of the attacking player guides the control of the interceptive actions of the defenders, we looked at the relationship between the tau of the honest (COM) and deceptive (upper trunk yaw) signals and the tau of the closure of the gap between the attacker and the defender (Figure 6). The tau-coupling model hypothesises that by keeping the action tau linked to the information or perception based tau then both gaps should close simultaneously.

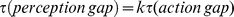



In the above equation the perception gap could be either the honest signal (COM displacement) or deceptive signal (upper trunk yaw) and was calculated as above, but this time with respect to the interception zone *after* the point of reorientation. The action gap was defined as the difference between the current COM of the attacker and the current COM of the defender and the tau of this gap was calculated as above. The k represents a coupling constant (Figure 6). Tau coupling analysis has previously been used to explain how to catch [23], [26] or strike [25] an object but not to intercept a moving person. Here we hypothesise that the experts will use the tau of the honest signal to guide their interceptive actions while the novices will use the tau of the deceptive signals (upper trunk yaw in this instance).

#### Participants

Twelve expert rugby players (M = 23.9 years; SD = 2.9 years) and 12 non-rugby players (M = 22.6 years; SD = 2.6 years) took part in the study.

#### Stimuli

The same deceptive and non-deceptive movements as those used in experiment 1 were again employed in experiment 2. The main differences, however, were that the displays were no longer cut off at different key moments in the movement. Instead the whole movement was presented in the head mounted display and players were free to choose when and how to act.

#### Task

Participants wore a stereoscopic HMD and backpack housing the control unit (to make it mobile) along with a head tracker. This gave an immersive, interactive experience in a virtual rugby environment where there was a 1∶1 mapping between displacement in the virtual world and displacement in the real world. The same virtual attacking movements (8 DM, 4 NDM) used in experiment 1, but with no occlusion, were pseudo-randomly presented five times in experiment 2 (total 60 trials). Participants again took on the role of defender and were asked to move to intercept or tackle the virtual player (Video S2). Player movement was recorded by placing 38 reflective markers on key anatomical landmarks on the player’s body. To obtain more accurate recordings the markers were placed on skin-tight sports’ clothing. Marker displacement was recorded in 3 dimensions at 120 Hz using six infrared Qualisys ProReflex motion capture cameras (Qualisys, Gothenburg, Sweden) (Figure 3 and Video S2).

#### Motion analysis

The external markers attached to the body of the participants were used to compute the different positions of the joint centres of the 12 different segments presented in the Zatsiorsky anthropometric table [36]. Each segment’s position (*G_i_*) was weighted by its mass (*m_i_*) to obtain the global COM position using the following formula:
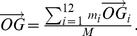
(3)where *O* is the origin of the reference frame, *G* the COM position and *M* the global mass of the body.

In the case of an early movement bias, the magnitude of the gap was obtained by computing the difference between the maximum COM displacement M/L in the wrong direction and the initial COM M/L position recorded before any movement was initiated. The beginning of the displacement in the wrong direction was taken as being the point when the COM M/L displacement velocity passed a 0.5 m/s threshold.

## Results

### Experiment 1: Perception Only

A mixed-design ANOVA that compared the percentage of correct responses averaged for each of the participant groups (between factor) across the 4 occlusion times (within factor) and for the two types of movement (deceptive (DM) and non-deceptive (NDM)) (within factor), showed that there was a significant main effect for type of movement with fewer correct responses for deceptive movements compared to non-deceptive movements (F_(1,208)_ = 318.90, p<.001, η^2^ = .03; Figure 1). Furthermore, a significant main effect for group further revealed that experts performed significantly better than novices (F_(1,208)_ = 118.96, p<.001, η^2^ = .01), particularly in the DM condition at T1 (Exp. M = 81.79% ±17.91% vs. Nov. M = 24.46% ±10.88%, p<.001), and as more information was made available (significant main effect for occlusion times; F_(3,208)_ = 777.80, p<.001, η^2^ = .04). This was the case for the deceptive rather than non-deceptive movements where a ceiling level was reached for the experts at T0 (M = 96.79%; sd = 6.68%) and the novices at T1 (M = 96.79%; SD = 3.16%).

We can use these findings to make predictions about how players (novice and expert) should respond when faced with a virtual side-stepping attacker. By analysing the dynamics of the relative movement of different parts of the body of the attacker with respect to the point of body reorientation we can see how biological motion influences judgements about the final running direction (Figure 1 and Figure 2). From Table 1 it can be seen that the highest R^2^ values were found for the tau COM (honest signal) in the expert group (R^2^ = .76) and for the tau upper trunk yaw (deceptive signal) in the novice group (R^2^ = .67). This strongly suggests that the experts were tuning into the honest signals while the novices were tuning into the deceptive signals. Furthermore the critical values (CV) that represent the point where the percentage of correct judgements equals 50%, suggest that the time when experts are tuning into the action relevant information specifying true running direction is much earlier than the novices (Expert CV = −183 ms; Novice CV =  −16 ms before reorientation for the COM signal). This further explains why expert performance was significantly better than novice performance particularly at times T0 and T1 (Figure 5).

### Experiment 2: Perception and Action

By analysing the players’ action responses we can see how the perceptual information is influencing the control of their actions. In other words we can quantify the extent to which players are ‘fooled’ by the deceptive movements. The results from the temporal occlusion paradigm used in experiment 1 (perception only) showed how novices made more errors in judging final running direction when compared to experts, particularly in the early parts of the movement. The critical values obtained from the regression analysis also suggested that novices were picking up information about the actual final running direction much later than the experts. If the perceptual information identified in experiment 1 is indeed guiding the control of the action (when and how to act), we would predict that the novices, who are tuning into the deceptive signals and anticipating the wrong running direction, would initiate their movements much earlier than experts.

**Figure 7:**
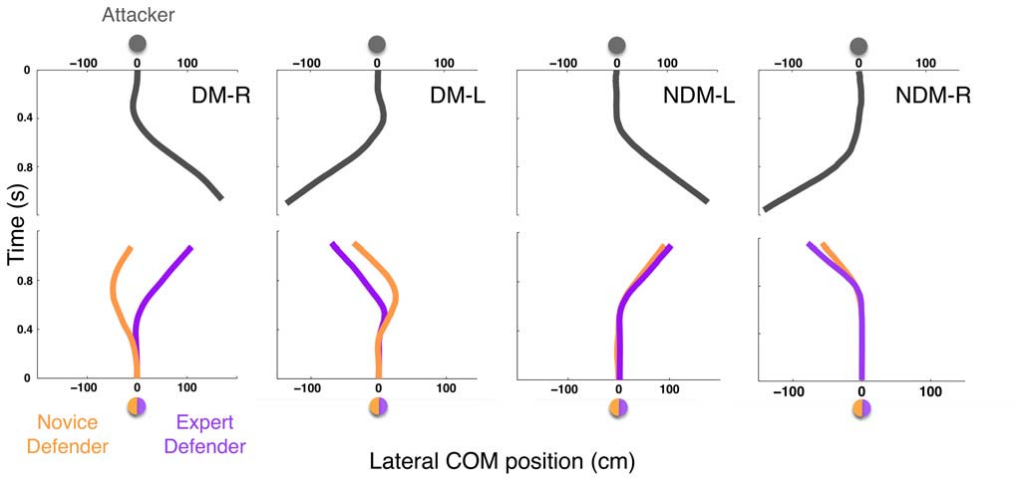
Effects of expertise on movement initiation and displacement. Four examples of how the virtual attacker’s movements (dark grey - DM-R (deceptive movement right), DM-L (deceptive movement left), NDM-L (non-deceptive movement left) and NDM-R (non-deceptive movement right)) influence the movements of an expert (purple) and a novice (yellow) defender. Displacements represent the lateral movement (cm) of the COM (centre of mass) over time (0 s corresponds to T0 in the Perception Only experiment – Figure 4). Note how the novice (yellow line) moves in the wrong direction and initiates his movements earlier than the expert in the DM conditions.

An analysis of the movement initiation times (ms) confirmed this and showed that experts waited significantly longer before moving to ‘intercept’ the virtual attacker when compared to novices (Experts M = 267.74 ms; SD = 36.18 ms vs. Novices M = 192.71 ms; SD = 63.82 ms) (t_(22)_ = 3.54; p = .002, d = 1.45) (Table 2). Furthermore, if the novices are tuning into the deceptive signals we would also predict that the number and amplitude of the initial movements in the wrong direction would be greater than the experts. Again the results showed that when confronted with a deceptive movement novices made a significantly greater percentage of movements in the wrong direction (movement biases) (M = 41.87%; SD = 20.53%) compared to the experts (M = 14.16%; SD = 9.8%) (t_(22)_ = 4.22, p<.001, d = 1.72). Furthermore these early movement biases were of a significantly greater amplitude for novices (M = 14.99 cm; SD = 2.68 cm)) compared to experts (M = 11.74 cm; SD = 3.81 cm) (t_(22)_ = 2.41; p = .025, d = .98) (Figure 7 and Table 2).

**Table 2:** Results from experiment 2 (Perception and Action).

	Experts		Novices	
	DM	NDM	DM	NDM
Initiation displacement (ms)	285.3±116.5	244±95.7	192.6±137.2	179.41±112.16
Number early bias (%)	14.16%	–	41.87%	–
Amplitude early bias (cm)	12,81±8.01	–	15.51±8.92	–
Performance - final distance Att./Def. (cm)	48.27±33.17	95.42±35.63	70.98±32.41	105.64±40.55

The table shows the time when the displacement was initiated (ms), the percentage of early bias trials, the amplitude of the early bias (cm) and the final distance between the attacker and the defender (cm- a performance related measure). The values are classified per group (expert/novice) for the DM condition only.

Finally as this is an interceptive task where the goal is to ‘intercept’ or block the attacking player, we looked at how the final distances between the virtual attacking player and the real defenders differed between our expert and novice groups. Again, the final distance between players was significantly smaller (t_(22)_ = 5.43; p<.001) for the experts (M = 49.2 cm; SD = 11.4 cm) compared to the novices (M = 70.8 cm; SD = 7.6 cm) indicating superior levels of task performance.

If the tau variables, highlighted in experiment 1, are being used to anticipate final running direction, we hypothesise that this perceptual informational variable (tau) is also being used to guide the defender’s interceptive action [23]. With this in mind we examined how the tau of the honest (COM) and deceptive signals (upper trunk yaw) *after* the reorientation point were correlated with the tau of the closing distance gap between the defending player’s COM and the attacking player’s COM (the tackle or interception zone; see Figure 6). The analysis between the perceptual information and the defending expert player’s movements showed a strong relationship (mean R^2^ = 0.94; SD = 0.05) between the tau of the COM (honest perceptual signal) and the tau of the distance gap between the defending player’s COM and the attacking player’s COM (action parameter) (Figure 6; Table 3). Furthermore, this relationship between the expert player’s action and the honest perceptual signal (tau COM) was significantly greater than the relationship between the expert player’s action and the deceptive perceptual signal (tau upper trunk yaw) (mean R^2^ = 0.77; SD = 0.12) (t_(11)_ = 3.95; p<.01). In contrast, the novices tended to use the deceptive signal (tau upper trunk yaw) to guide their actions (tau distance gap between defender’s COM and attacker’s COM) (mean R^2^ = 0.76; SD = 0.09) more than the honest signal (tau COM) (mean R^2^ = 0.64 SD = 0.21). When comparing the relationship between signal use and the player groups, experts were found to have a significantly stronger correlation between the honest perceptual information and their unfolding action compared to the novices (t_(22)_ = 4.7; p<.001) (Table 3).

**Table 3:** Results showing how perception influences action.

	Experts		Novices	
	DM	NDM	DM	NDM
**Honest Signal**R^2^ - Tau Attacker's COM/Tau gap distance Att-def	0.94±0.05	0.6±0.13	0.64±0.21	0.51±0.1
**Deceptive Signal**R^2^ - Tau Attacker's Upper Trunk Yaw/Tau gapdistance Att-def	0.77±0.12	0.19±0.14	0.76±0.09	0.15±0.13

The results in the table show the relationship between the perceptual information (tau) and the corresponding action (tau) for novices and experts in the DM condition. Note how the experts’ movements to intercept the attacking player are more coupled to the tau of their COM an honest signal than the upper trunk yaw a deceptive signal. The novices on the other hand tend to use a deceptive signal (upper trunk yaw) more than the honest signal (COM displacement).

## Discussion

The objective of this study was twofold. Firstly, we aimed to explore the nature of the information used by experts and novices to predict the final running direction of an opponent. In other words we wanted to understand how prospective information embedded in the body-based kinematics is used to judge an opponent’s final running direction. Secondly, we analysed the relationship between perceptual expertise and the dynamics of the ensuing action in order to see *how* perceptual information informs decisions about *when* and *how* to act. Our tau-model for detecting deceptive movement accurately predicted expert and novice differences in both recognising (perceptual judgements) and responding (action responses) to deceptive movement.

With respect to perceptual skills, we show that experts are more attuned to honest signals (e.g. COM displacement M/L) that specify future running direction whilst novices are more attuned to deceptive signals (e.g. head yaw, upper trunk yaw and OF displacement M/L) that do not specify future running direction. In addition, the large differences found in the CVs that indicate when the information is being picked up for the tau variable, suggest a greater sensitivity to relevant final running direction information in experts compared to novices. In other words, for a given parameter, experts are able to get a majority of correct responses (>50%) earlier than the novices. Consequently, we can suppose that they are able to accurately anticipate the outcome of an attacking movement with less information.

In terms of the action responses, the results highlight the fact that experts wait significantly longer than novices before initiating a displacement to ‘intercept’ the virtual attacker. This delay translates into significantly fewer movement errors in the wrong direction. Any movements that do occur in the wrong direction are also of a significantly lower amplitude than those made by the novices. This can again be explained by the experts’ superior ability to tune into the dynamics of body based information that specifies the true running direction (namely tau-COM), which would minimise the number of movement errors in the wrong direction.

Using the metaphor of evolutionary theory, a selective advantage is gained if an opponent tunes into the honest signals and ignores the deceptive signals [21]. This is exactly what we show here. The predictive power of the invariants identified in the dynamics of body based movement used to signal deception suggests that the novices will make more errors than the experts. These results support the need for perceptual training so that the invariance associated with key honest signals (e.g. the dynamics of COM displacement) are recognised and used to guide future action [37].

Previous studies, using temporal occlusion paradigms, have also shown an expert advantage in picking up early information that specifies the outcome of an action [15], [22], [38]. In these cases the superior perceptual ability demonstrated by experts often related to the ability to ‘read’ the kinematics of the movement. The authors suggested that it is the perceptual experience of the experts that explains why they are more proficient at detecting deceptive movement. In other words it is their superior task-relevant visual experience that counts [15], [38].

Although the perceptual experience hypothesis explaining expertise has focused on the ability to make superior perceptual judgements [15], [22], it has tended to neglect the role that prospective, perceptual-based information specified through the unfolding pattern of body movement, plays when making anticipatory judgements. It also fails to address how prospective information influences or guides decisions about when and how to act. The solution presented here addresses both these shortcomings. Through the analysis of successful deceptive movements, we have shown how the tau of honest or deceptive signals embedded in these movements can be picked up and used by the perceiver to anticipate the attacker’s final running direction. These perceptual variables can in turn influence both the action choices made by the defenders (i.e. when and how to act) and the overall control of the action to arrive in the right place at the right time (perceptual guidance of action).

An alternative explanation for expertise in both perception and action domains is the common coding theory [39]–[41]. This theory suggests that there is a mapping between observed action and the observer’s own motor repertoire (e.g. a side step in rugby), suggesting a common neural code for both perception and action. Studies have shown that if the observer does not have the appropriate motor repertoire that corresponds to the observed action, then the neural resonance is greatly diminished or non-existant [42]. These studies have often focused on action recognition and have not attempted to identify the information that is embedded in different movement kinematics associated with different actions. In this paper we show that the temporal dynamics of perception and action based events could be coded using a common currency that is time based (i.e. tau). We show that expertise is more related to tuning into relevant body-based information that can be used to anticipate and guide actions. Future work should try and understand how the temporal dynamics of actions are coded so we can further understand the neural links between perception and action.

Finally, in order to maximise the effects of movement based deception it is prudent to try to detract attention away from the honest signals that specify true action intention and move it towards the deceptive signals that do not. From the point of view of the person or animal being preyed upon, emphasising deceptive signals could give further selective advantage [43]. One such example is that of feral pigeons who have a white patch on the base of their tail. Evolutionary biologists have shown how this colour contrast between the lower back/tail region and the rest of the body detracts a falcon’s attention away from the wing movement, or ‘honest’ signals, that signify the beginning of an evasive roll [44]. This visual distraction has been shown to significantly affect their rates of survival. Similarly in sport, it could be suggested from this study that a player who wears fluorescent coloured boots and/or has contrasting colours on the shoulders or upper part of his/her shirt would attract a player’s visual attention away from the honest signals and more towards the deceptive signals during a side step (e.g. out-foot placement, upper trunk yaw) giving that player more of a competitive advantage. Future work should investigate the role of visual attention in tuning into deceptive and honest signals.

To conclude we have shown how experts appear to tune into the dynamics of honest signals that specify true running direction while novices tend to use the deceptive signals that do not. Furthermore we have shown how the tau of the Centre of Mass (an honest signal) can explain the superior performance in the expert group while the tau of the upper trunk yaw (a deceptive signal) can explain the poorer performance in the novice group. By using state of the art immersive interactive virtual reality technology we were also able to show how the perceptual information the players were tuning into also guided their interceptive actions and influenced decisions about when and how to act (perception and action experiment). Because experts were picking up relevant information from the honest signals they made fewer action based errors than the novices and were able to guide their actions to successfully intercept the attacking player. The implications of the findings extend far beyond the side-step in rugby and provide a framework for understanding how perceptual information picked up from the movement kinematics of different body segments can be used to anticipate and guide a future course of action.

## Supporting Information

Video S1 **Deceptive Movement Stimulus creation.** The different steps involved in the animation process are presented in this video. The video shows how the virtual animations come from real-life motion capture of 1 vs. 1 duels between a real attacker and defender. This motion capture data are then used to animate a skeleton which forms the basis of the movement of the realistic character animated in the virtual rugby environment.
(MOV)

Video S2 **Experiment 1 - Perception only.** This video highlights the design of the perception only experiment. The first part shows the immersive interactive environment with the virtual rugby pitch, the virtual attacker and the head tracking solution used to update the participant’s viewpoint in real time in the virtual environment. The second part of the video illustrates the task the participants were asked to perform, namely, judge the final running direction of a virtual attacker at different occlusion times (times T0 and T3 are shown in the video).
(MOV)

Video S3 **Experiment 2 - Perception & Action.** The Perception and Action experiment is illustrated in this video. Firstly we see an example of a virtual attacker’s side-step, without any occlusion. Next we see a real participant wearing the HMD, head tracker and backpack performing the experiment. The whole system allows the participant to move freely and to attempt to intercept the attacker as if they were performing a real 1 vs. 1 duel in rugby. Note, that the participant is also wearing 38 reflective markers so that his full body movement is captured for later analysis. The last part of the video shows what the recorded motion capture looks like after reconstruction.
(MOV)
